# Glucagon-Like Peptide-2 Ameliorates Lipid Metabolism in Metabolic Dysfunction–Associated Steatotic Liver Disease Through the Adiponectin-Adiponectin Receptor-Mediated AMPK/PPARα Pathway

**DOI:** 10.33549/physiolres.935711

**Published:** 2026-02-01

**Authors:** Shu-Juan ZHANG, Ke XU, Feng ZHU, Jun-Guo CHEN, Ying ZHANG, Yi-Qun TENG, Fang-Fang SHEN

**Affiliations:** 1 Department of Pediatrics, The Second Hospital of Jiaxing, Jiaxing, Zhejiang, China; 2 Department of Pediatrics, Xianju People’s Hospital, Zhejiang Southeast Campus of Zhejiang Provincial People’s Hospital, Affiliated Xianju’s Hospital, Hangzhou Medical College, Xianju, Zhejiang, China

**Keywords:** Adiponectin, AMPK, Glucagon-like peptide-2, Metabolic dysfunction-associated steatotic liver disease, PPARα

## Abstract

The aim of this study was to investigate the mechanisms by which glucagon-like peptide-2 (GLP-2) improves metabolic dysfunction-associated steatotic liver disease (MASLD) induced by free fatty acids (FFAs) in HepG2 cells, with a focus on the regulation of the adiponectin (ADPN) signaling axis and the downstream AMP-activated protein kinase (AMPK)/peroxisome proliferator-activated receptor α (PPARα) pathway. An MASLD model was established in HepG2 cells by FFA exposure. Following GLP-2 treatment, improvements in lipid metabolism were evaluated using the Cell Counting Kit-8, Oil Red O staining, and biochemical assays. Differential gene expression was examined using RNA sequencing, and potential mechanisms were evaluated through Gene Ontology enrichment and Kyoto Encyclopedia of Genes and Genomes pathway analysis. Western blotting and reverse transcription polymerase chain reaction (RT-PCR) were performed to assess the expression of key molecular components within the signaling pathway. FFA treatment led to significant lipid accumulation in HepG2 cells, whereas GLP-2 reduced intracellular lipid droplet formation (p <0.01) and decreased triglyceride and total cholesterol levels in a dose-dependent manner (p <0.05). KEGG enrichment analysis indicated that GLP-2 acted on t he adipokine, AMPK, and P PARα pathways. Western blotting and RT-PCR confirmed that GLP-2 restored protein expression (p <0.01) and mRNA expression (p <0.05) of adiponectin receptor 1, adiponectin receptor 2, and downstream signaling molecules AMPK and PPARα in FFA-treated HepG2 cells. GLP-2 alleviated FFA-induced hepatocyte steatosis by modulating the AMPK/PPARα pathway through the regulation of ADPN and its receptors. These findings provide a theoretical foundation for the potential use of gut hormones in the treatment of MASLD.

## Introduction

Metabolic dysfunction-associated steatotic liver disease (MASLD) is a chronic liver condition strongly associated with obesity and has emerged as a leading cause of liver-related morbidity and mortality worldwide [1,2]. The global prevalence is estimated to be 32.4 %, with an annual incidence of 46.9 cases per 1000 person-years [3]. Among individuals who are overweight, the prevalence reaches 69.99 % [4]. The hallmark pathological feature of MASLD is excessive lipid accumulation within hepatocytes, frequently accompanied by metabolic disturbances such as insulin resistance (IR), obesity, and type 2 diabetes [5,6]. Despite increasing recognition of MASLD, the precise mechanisms underlying disease progression remain unclear, and effective targeted therapies are limited [7,8].

Glucagon-like peptide-2 (GLP-2) is a gastrointestinal hormone primarily secreted by enteroendocrine L cells, with additional synthesis occurring in the gastrointestinal tract, liver, adipose tissue, and central nervous system. Its secretion is predominantly stimulated by nutrients, particularly fats and glucose [9]. The primary physiological functions of GLP-2 include the promotion of intestinal mucosal growth and the enhancement of nutrient absorption [10]. Beyond these roles, GLP-2 contributes to maintaining a positive energy balance, delaying the onset of IR, regulating hepatic lipid homeostasis, and modulating lipid metabolism [11]. GLP-2 exerts its biological functions in the intestine and other tissues through the GLP-2R [12,13]. Neuronal glucagon-like peptide-2 receptor (GLP-2R) signaling influences intestinal lipid absorption, hepatic glucose metabolism, and feeding behavior through the gut-brain axis [14–16]. In murine models, GLP-2R deficiency results in impaired postprandial glucose tolerance and hepatic IR [17]. With increasing recognition of its biological and pharmacological functions, GLP-2 has gained attention as a potential therapeutic target for modulating the gut-liver axis metabolism [18,19]. However, the mechanisms through which GLP-2 regulates this axis in MASLD remain poorly defined.

Adiponectin (ADPN) is an adipokine that enhances hepatic lipid oxidation and reduces inflammation by activating adiponectin receptor 1 (AdipoR1) and adiponectin receptor 2 (AdipoR2), which in turn regulate the AMP-activated protein kinase (AMPK)/peroxisome proliferator-activated receptor α (PPARα) pathway [20]. In patients with MASLD, serum ADPN levels are significantly reduced and inversely correlated with disease severity [21]. AdipoR1 primarily activates the AMPK pathway, thereby reducing hepatocyte lipid accumulation and promoting fatty acid oxidation, whereas AdipoR2 activates the PPARα pathway, enhancing lipid metabolism and modulating inflammatory responses [22,23]. High-molecular-weight ADPN oligomers are especially effective in suppressing hepatic glucose production and improving metabolic dysfunction. These oligomers exert protective effects in metabolic dysfunction-associated steatohepatitis (MASH) and liver fibrosis by mitigating IR [24]. Although GLP-2 has demonstrated beneficial effects on hepatic lipid metabolism, its relationship with the ADPN signaling pathway has not been fully clarified. Specifically, it remains uncertain whether GLP-2 improves lipid metabolism disturbances in MASLD through modulation of the ADPN signaling axis. A recent clinical study by our group demonstrated that elevated serum GLP-2 levels are significantly associated with insulin resistance and dyslipidemia in children with obesity and MASLD, identifying it as a potential biomarker [25]. While this human study highlighted the clinical relevance of GLP-2 in MASLD pathophysiology, the precise molecular mechanisms by which GLP-2 exerts its effects on hepatocytes remain unclear.

The present study investigated the effects of GLP-2 on free fatty acid (FFA)-induced hepatocyte lipid metabolism disturbances, with a focus on the regulatory role of the ADPN/AdipoR-AMPK/PPARα signaling axis. These findings provide a theoretical basis for the development of gut-liver axis-targeted therapies for MASLD.

## Materials and Methods

### Cell culture and steatosis model establishment

The HepG2 human hepatocellular carcinoma cell line was obtained from the National Collection of Authenticated Cell Cultures. Cells were maintained in Dulbecco’s modified Eagle medium (DMEM, high glucose; Gibco) supplemented with 10 % fetal bovine serum (FBS; Gibco) and 1 % penicillin-streptomycin (Beyotime, Shanghai, China). Cultures were incubated at 37 °C in a humidified atmosphere containing 5 % CO_2_. Cells were passaged every 2 to 3 days using 0.25 % trypsin (Sigma). To establish the steatosis model, HepG2 cells were seeded at 1×10^5^ cells per well in six-well plates and incubated for 24 h until cell adhesion was confirmed. Steatosis was induced by exposing cells to an FFA/bovine serum albumin (BSA; Sigma-Aldrich) complex solution (combined concentration 1 mM) for 24 h. The modeling solution consisted of a 2:1 mixture of palmitic acid (PA) (0.25 mM) and oleic acid (OA) (0.5 mM) (SYSJ-KJ006, Kunchuang, Xian, China) [26]. Control cells were treated with BSA-containing medium only.

### Cell viability assay

Cells were plated in 96-well plates and divided into treatment groups receiving 10, 100, 1000, 2000, or 4000 nmol/l GLP-2. A control group (Con) and a blank group (medium without cells or drug) were included. Each group contained six replicates. After incubation for 24 and 48 h, the culture medium was discarded, and 10 μl of Cell Counting Kit-8 (CCK-8; Beyotime, Shanghai, China) solution with 90 μl of medium were added per well. Following incubation for 2 h, absorbance was measured at 450 nm to assess cell viability.

Cell viability = [(OD value of the treatment group - OD value of the blank group) / (OD value of the control group - OD value of the blank group)] × 100 %.

### Experimental grouping and GLP-2 intervention model establishment

Steatosis model cells were cultured with FFA-free medium containing varying concentrations of GLP-2 (1–33, Bachem AG, Bubendorf, Switzerland) at 10, 100, and 1000 nmol/l. Equal volumes of FFAs-free complete medium were added to the blank control and FFA-treated groups. Cells were incubated for 24 h before further experiments. The experimental groups included: blank control group (control), FFA-treated group (FFAs), and GLP-2 + FFAs.

### Oil red O staining

At the conclusion of treatment, HepG2 cells cultured in six-well plates were rinsed with phosphate-buffered saline (PBS; HyClone), fixed in 40 g/l paraformaldehyde (Boster Biotechnology Co., Ltd., Wuhan, China) for 30 min at room temperature, and washed with PBS. Cells were immersed in 600 ml/l isopropanol for 1 min, followed by staining with 5 g/l Oil Red O solution (Solarbio, Beijing, China) prepared in 600 ml/l isopropanol for 15 min. After three washes with distilled water, cells were mounted with glycerol jelly mounting medium (Beyotime, Shanghai, China). Lipid accumulation was evaluated using a light microscope (Olympus, Tokyo, Japan), and images were captured. Using ImageJ software (National Institutes of Health, Bethesda, Maryland, USA) to analyze Oil Red O-stained area and optical density, the Oil Red O-positive stained area and integrated density (Integrated Density) in each field of view were quantified by setting a unified threshold. The measured values were further normalized using the number of DAPI-stained nuclei to correct for differences in cell density (Parazacco spilurus subsp. spilurus). Ultimately, the degree of lipid accumulation was expressed as the average lipid accumulation per cell.

### Biochemical assays

Cell culture supernatants were collected and centrifuged at 3000 rpm for 10 min. The supernatant was retained for analysis. Triglyceride (TG) and total cholesterol (TC) concentrations were measured using commercial kits (Nanjing Jiancheng Bioengineering Institute, China) according to the manufacturer’s instructions. Absorbance was measured using a microplate reader (BioTek), and concentrations were determined from standard curves.

### Transcriptome sequencing

Cells from each treatment group were collected, frozen in liquid nitrogen, and stored at −80 °C. Total RNA was extracted, and libraries were prepared for sequencing on the Illumina NovaSeq 6000 platform. Transcriptome analysis was performed by Shenzhen BGI Genomics Co., Ltd. (Shenzhen, China). Differentially expressed genes (DEGs) were identified using DESeq2 (v1.4.5) with a Q value threshold of <0.25. Gene Ontology (GO) and Kyoto Encyclopedia of Genes and Genomes (KEGG) enrichment analyses were conducted using the phyper function in R based on hypergeometric tests. Genes with Q values <0.25 were considered significantly enriched. Transcriptome data were normalized using R software.

### ELISA

Conditioned medium from each well was centrifuged (500× g, 5 min) to remove cell debris. Total adiponectin (Abcam, cat. ab99968) and HMW adiponectin (CUSABIO, cat. CSB-E07270h) were measured in duplicate according to the manufacturers’ instructions and normalized to total cellular protein.

### Western blot analysis

Protein extracts were isolated from HepG2 cells using a protein lysis kit (Beyotime, Shanghai, China), and protein concentrations were determined by the BCA method (BCA Protein Assay Kit, Beyotime, China) to ensure consistent loading amounts. Subsequently, equal amounts of protein samples were separated on 10 % SDS-PAGE gels. Proteins were transferred to polyvinylidene fluoride (PVDF) membranes, blocked with 5 % skim milk for 1 hour, and incubated overnight at 4 °C with primary antibodies against AdipoR1 (Monoclonal, Proteintech, China, cat. no. 66619-1-lg, 1:2000, 43 kDa), AdipoR2 (Polyclonal, Bioss Antibodies, China, cat. no. bs-0611R, 1:1000, 44 kDa), AMPK (Polyclonal, Proteintech, China, cat. no. 10929-2-AP, 1:5000, 63 kDa), and PPARα (Monoclonal, Proteintech, China, cat. no. 66826-1-lg, 1:2000, 53 kDa). β-actin was used as the internal reference. After equilibrium at room temperature for 30 min, membranes were washed and incubated with an HRP-conjugated secondary antibody (1:1000 in 5 % skim milk) for 90 min. After washing, membranes were developed and imaged using a Bio-Rad multicolor gel imaging system. Band intensity was quantified using ImageJ, and protein expression levels were normalized to β-actin.

### Quantitative reverse transcription polymerase chain reaction (RT-PCR) analysis

Primers for *ADIPOR1*, β-actin, and other genes were designed using Primer Premier 5.0. Total RNA was extracted using the TRIzol reagent, and cDNA was synthesized for gene expression analysis using a Bio-Rad iQ5 real-time PCR system. Reaction conditions included an initial denaturation at 95 °C for 10 min, followed by 40 cycles of 95 °C for 10 seconds, 62 °C for 20 s, and 72 °C for 20 s. A melting curve program was applied from 40 °C to 99 °C at 0.1 °C/s with continuous fluorescence detection. Relative gene expression was calculated using the 2^−ΔΔCt^ method, with β-actin as the internal reference. Primer sequences and parameters are presented in Table 1.

### Statistical analysis

Statistical analysis was performed using SPSS software (version 21.0; SPSS Inc., Chicago, IL, USA). Homogeneity of variance was tested. For data with homogeneous variances, one-way analysis of variance (ANOVA) followed by Fisher’s least significant difference (LSD) test was applied. For non-homogeneous variances, the Kruskal-Wallis test was used. If significant differences were identified, the Mann-Whitney U test was conducted for pairwise comparisons. The significance threshold (α=0.05) was adjusted using the Bonferroni correction. Results are expressed as mean ± standard deviation (SD). All statistical tests were two-tailed, with significance defined as *p*<0.05.

## Results

### Effect of GLP-2 on cell viability

**Fig. 1:**
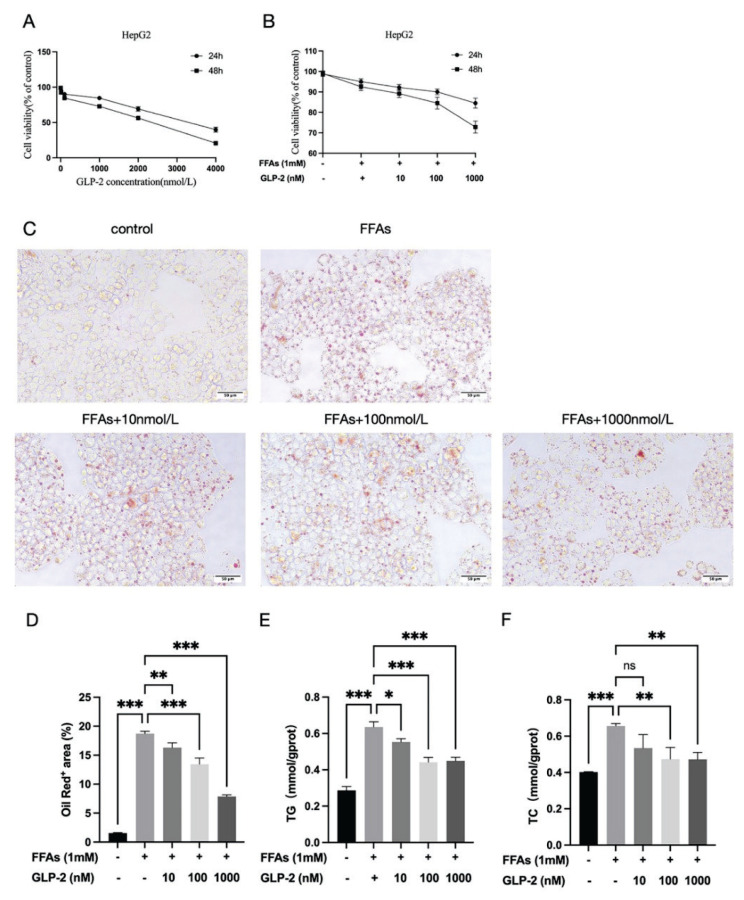
Effect of GLP-2 administration on FFA-induced lipid accumulation in HepG2 cells. (**A**) Effect of GLP-2 on HepG2 cell viability (mean ± SD, n=6). Cells were divided into five groups: control, FFAs, 10 nmol/l GLP-2 + FFAs, 100 nmol/l GLP-2 + FFAs, and 1000 nmol/l GLP-2 + FFAs. (**B**) Survival rate of HepG2 cells in each group (mean ± SD, n=6). (**C**) Oil Red O staining of intracellular lipid accumulation (scale bar = 50 μm). Representative images are presented. (**D**) Quantification of intracellular lipid content based on Oil Red O staining. (**E**) Intracellular TG levels assessed using biochemical assay kits. (**F**) Intracellular TC levels assessed using biochemical assay kits. All experiments were conducted independently three times, with results presented as mean ± SD. ns: not statistically significant; ** p <0.01, *** p<0.001.

Cell viability decreased after 24 and 48 h of treatment with varying GLP-2 concentrations compared to the control group. At 24 h, viability remained above 80 % at concentrations below 2000 nmol/l but fell below 80 % at higher concentrations (Fig. 1A). Based on these findings, 10 and 1000 nmol/l GLP-2 were selected as the low- and high-dose treatments, respectively, and a 24-hour treatment duration was used in subsequent experiments. At the end of all experimental groups, cell viability was measured again. Compared with the FFAs group, the GLP-2 treatment group (10–1000 nmol/l) exhibited cell viability above 80 % with no significant toxicity (Fig. 1B).

### GLP-2 improved hepatocyte steatosis

To investigate the mechanisms by which GLP-2 reduces lipid accumulation, HepG2 cells were treated with FFAs and subsequently exposed to different GLP-2 doses for 24 hours. While FFA treatment significantly increased intracellular lipid accumulation, GLP-2 reduced lipid droplets in a dose-dependent manner (*p*<0.01; Figs 1C-D). In addition, GLP-2 significantly suppressed FFA-induced TG and TC accumulation (*p*<0.01; Fig. 1E–F). These results demonstrate that GLP-2 effectively restores lipid metabolism homeostasis in HepG2 cells.

### Transcriptome sequencing analysis identified the core mechanism of GLP-2 in regulating MASLD

#### GLP-2 activated a distinct transcriptional regulatory network

**Fig. 2:**
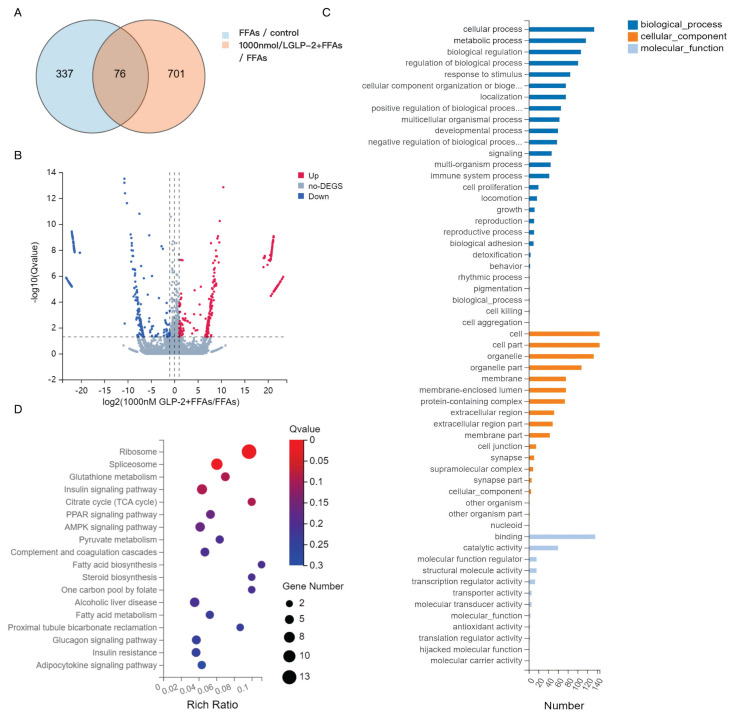
Transcriptomic analysis of GLP-2 treatment in HepG2 cells. (**A**) Venn diagram presenting 76 overlapping DEGs between the FFAs vs. control and the 1000 nmol/l GLP-2 + FFAs vs. FFAs groups. (**B**) Volcano plot demonstrating DEGs between the 1000 nmol/l GLP-2 + FFAs and FFAs groups. Red: upregulated genes; green: downregulated genes; gray: non-significant genes. (**C**) GO enrichment analysis (n=3) depicting gene distribution across biological processes, cellular components, and molecular functions in the GLP-2-treated group. (**D**) KEGG pathway enrichment analysis (n=3) indicating activation of adipokine signaling, AMPK, and PPARα pathways in the GLP-2-treated group.

Venn diagram analysis revealed 76 DEGs common to both the FFA-treated pathological group (FFAs vs. control) and the GLP-2 rescue group (1000 nmol/l GLP-2 + FFAs vs. FFAs) (Fig. 2A). Notably, 701 DEGs were uniquely regulated by GLP-2, indicating its activation of novel protective mechanisms beyond those associated with the disease state.

#### GLP-2 dose-dependent gene regulation

RNA-seq analysis revealed substantial differential gene expression between GLP-2-treated and FFA-treated groups. Volcano plots demonstrated marked upregulation and downregulation of various genes.

A comparison between the high-dose GLP-2 treatment group (1000 nmol/l GLP-2 + FFAs) and the FFA group identified 777 DEGs (FDR<0.05, |log^2^ fold change|>1), of which 71 genes were significantly upregulated and 81 were significantly downregulated (|log^2^FC|>10). The volcano plot (Fig. 2B) illustrated extensive transcriptional reprogramming, with key lipid metabolism genes occupying both extremes. Upregulated genes were primarily associated with lipid clearance (e.g., fatty acid oxidase) and cytoprotective responses, whereas downregulated genes were predominantly involved in lipid synthesis (ACACA, fatty acid synthase [FASN], stearoyl-CoA desaturase [SCD]), gluconeogenesis (PEPCK), and pro-inflammatory signaling (TNF-α). Collectively, these findings indicate that GLP-2 mitigates hepatic fat accumulation by enhancing lipid degradation while suppressing lipogenic and pro-inflammatory pathways.

#### GO enrichment and KEGG pathway enrichment verification of core mechanisms

GO enrichment analysis indicated that GLP-2 treatment primarily targeted cellular metabolic processes, influencing structural and functional pathways through protein-binding and catalytic activities. As presented in Fig. 2C, biological processes dominated the enriched gene sets in the 1000 nmol/l GLP-2 + FFA group, with cellular and metabolic processes such as AMPK signaling regulation and lipid oxidation being most prominent. These results indicate that GLP-2 exerts its effects on MASLD by addressing both cellular dynamics and metabolic imbalance. Cellular component analysis revealed enrichment in cell and cell part categories, emphasizing the central role of hepatocyte integrity. Molecular function analysis highlighted binding and catalytic activity as dominant categories, underscoring the importance of protein interaction networks and enzymatic regulation in the “hepatocyte injury-metabolic disturbance-protein dysregulation” mechanism underlying GLP-2’s protective role in MASLD.

KEGG pathway enrichment analysis further identified core metabolic regulatory pathways involved in GLP-2’s intervention (Fig. 2D). DEGs were enriched in pathways related to fatty acid and lipid metabolism. Notably, the PPARα signaling pathway (Rich Ratio = 0.05, Q=0.16) and the AMPK signaling pathway (Rich Ratio = 0.04, Q=0.16) were synergistically activated, promoting fatty acid oxidation and lipid clearance. Although the adipocytokine signaling pathway did not reach statistical significance (Rich Ratio = 0.05, Q=0.30), its interaction with AMPK/PPARα indicated a role in enhancing insulin sensitivity. Together, these findings indicate that GLP-2 improves MASLD pathology primarily through activation of the AMPK–PPARα axis.

### GLP-2 treatment partially restored FFA-induced downregulation of ADPN, AdipoR1/R2, and their downstream signaling in HepG2 cells

Reduced ADPN levels exacerbate hepatic fat accumulation and inflammation. KEGG analysis indicated that GLP-2 may exert therapeutic effects on MASLD through modulation of adipokine signaling. To validate this hypothesis, the impact of GLP-2 on the adipokine-AMPK/PPARα signaling axis was examined in the FFA-induced HepG2 cell model of MASLD.

#### GLP-2 specifically increases the secretion of HMW adiponectin in HepG2 cells

The effect of GLP-2 treatment on adiponectin expression in HepG2 cells was detected by ELISA. Compared with the control group, FFAs significantly inhibited the secretion level of HMW adiponectin (*p*<0.01), while the secretion of total adiponectin was not inhibited (*p*>0.05). GLP-2 intervention increased the secretion levels of total adiponectin and HMW adiponectin in HepG2 cells (*p*<0.01).

#### Western blot quantitative data confirmed the proteinlevel regulatory mechanism of the adipokine pathway

**Fig. 3:**
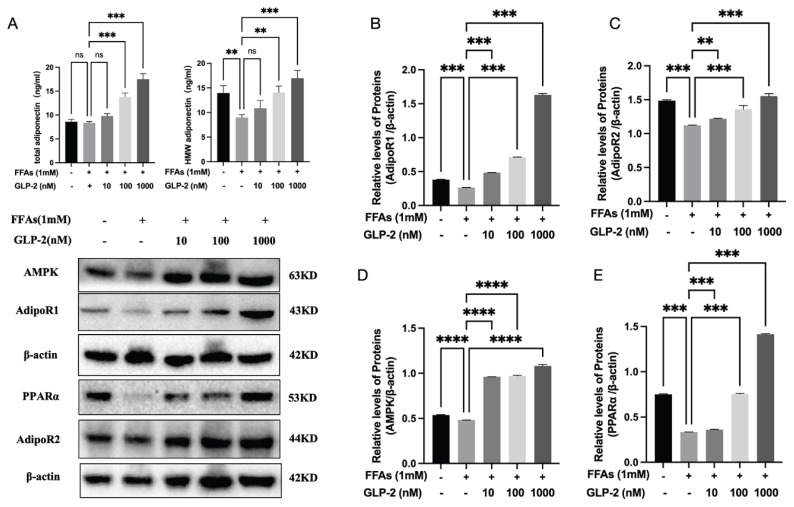
GLP-2 treatment reversed FFA-induced downregulation of ADPN, AdipoR1, AdipoR2, and associated downstream proteins in HepG2 cells. (**A**) ELISA detects the expression level of adiponectin in cell culture supernatant, (**B**) AdipoR1, (**C**) AdipoR2, (**D**) AMPK, and (**E**) PPARα protein expression levels. Data are presented as mean ± SD (n=3). Ns: not statistically significant. * p <0.05, ** p<0.01, *** p <0.001.

Western blotting was performed to evaluate protein expression of AdipoR1/R2, and their downstream effectors. As presented in Fig. 3, FFA treatment significantly reduced AdipoR1/R2, AMPK, and PPARα protein levels compared to the control group, whereas GLP-2 treatment restored their expression (*p*<0.01). These protein-level changes were consistent with the KEGG enrichment findings, supporting the hypothesis that GLP-2 modulates adipokine signaling in MASLD.

#### RT-PCR data confirmed transcriptional activation of the adipokine pathway

**Fig. 4:**
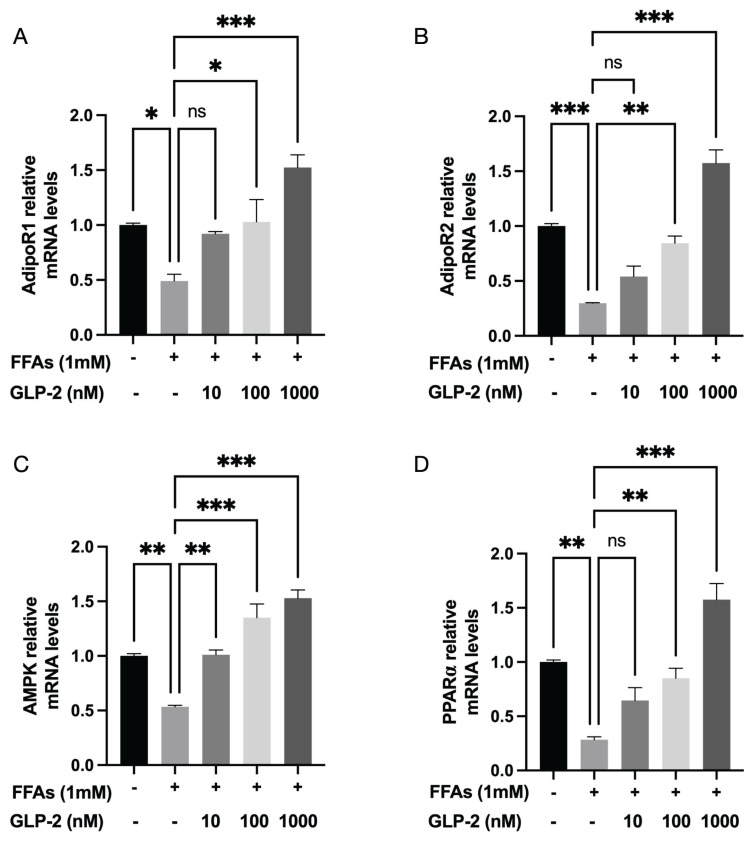
GLP-2 treatment reversed FFA-induced downregulation of AdipoR1, AdipoR2, and downstream signaling gene expression in HepG2 cells. mRNA expression levels of (**A**) ADIPOR1, (**B**) ADIPOR2, (**C**) PRKAA (AMPK), and (**D**) PPARA (PPARα). Data are presented as mean ± SD (n=3). ns: not statistically significant. * p <0.05, ** p<0.01, *** p<0.001.

RT-PCR analysis of AdipoR1/R2 mRNA expression in the FFA-induced HepG2 cell model of MASLD (Fig. 4) indicated that FFA treatment significantly reduced AdipoR1/R2 expression compared with the control group (*p*<0.01). Treatment with GLP-2 restored AdipoR1/R2 mRNA levels, with statistically significant differences observed (*p*<0.01). Expression of genes involved in downstream signaling pathways of *ADPN* receptors was also evaluated. FFA exposure led to a significant downregulation of *PRKAA* (AMPK)*K* and *PPARA* (PPARα) mRNA expression in HepG2 cells (*p*<0.01), consistent with western blot results. GLP-2 treatment partially reversed this effect, significantly increasing *PRKAA* and *PPARα* mRNA expression (*p*<0.05).

These functional experiments confirmed that GLP-2 activated AMPK phosphorylation by upregulating ADPN and its receptors AdipoR1/R2, which promoted PPARα nuclear translocation and improved FFA-induced lipid metabolism disturbances in HepG2 cells. This finding supports the synergistic regulation of the AMPK and PPARα pathways as indicated by KEGG enrichment analysis. Although restoration of AdipoR1/R2 function after GLP-2 treatment did not lead to significant changes throughout the entire adipokine signaling pathway, it effectively enhanced metabolic function by selectively activating the key downstream effectors AMPK and PPARα.

## Discussion

MASLD and its progressive stage, MASH, have emerged as significant global public health concerns. The underlying pathological mechanisms involve multiple interacting factors, including dysregulated lipid metabolism, IR, and chronic inflammation [27–29]. Current treatment options for MASLD remain limited, primarily relying on lifestyle modification and management of metabolic syndrome [30]. Therefore, identifying novel therapeutic targets and pharmacological interventions has substantial clinical relevance. Endogenous GLP-2 has been proposed as a potential protective factor against hepatic metabolic disorders, as it improves IR and supports hepatic lipid homeostasis [31–33]. However, the precise molecular mechanisms through which GLP-2 regulates liver lipid metabolism remain unclear. Recent studies have highlighted the unique role of GLP-2 in modulating the gut-liver axis. The present findings indicate that GLP-2 alleviates hepatocellular lipid metabolism disturbances by activating the ADPN signaling axis and the AMPK/PPARα pathway, thereby providing a foundation for the development of peptide-based drugs targeting the gut-liver axis [34].

In the FFA-induced HepG2 cell model, GLP-2 significantly improved MASLD-related pathology. FFA treatment markedly elevated lipid content in HepG2 cells, whereas GLP-2 reduced intracellular lipid droplet accumulation in a dose-dependent manner (*p*<0.01) and inhibited the accumulation of TG and TC (*p*<0.05). These findings indicate that GLP-2 ameliorates disturbances in hepatocellular lipid metabolism. In addition, GLP-2 regulates lipid metabolism through multiple mechanisms, including modulation of postprandial lipid absorption and lipid release during nutrient uptake [35]. This regulatory function is critical for maintaining lipid homeostasis and energy balance. In murine models, inhibition of the GLP-2R under high-fat diet (HFD) feeding leads to significant dyslipidemia and exacerbates hepatic injury, including steatosis [36]. The expression of GLP-2R in hepatic stellate cells further underscores its role in lipid regulation. In *Glp2r^−/−^* mice, hepatic lipid accumulation is increased, insulin sensitivity is impaired, and glucose metabolism deteriorates [37]. Furthermore, a long-acting GLP-2R agonist reduced body weight, liver weight, hepatic lipid deposition, inflammation, and fibrosis in a murine model of MASLD [38]. Collectively, these findings indicate that GLP-2 and its receptor function as protective factors in liver disease and represent potential therapeutic targets for MASLD.

Transcriptome sequencing analysis in this study demonstrated that GLP-2 improved MASLD pathology by modulating 701 specific targets. The adipokine and AMPK/PPARα pathways were identified as dominant, synergistically activating lipid metabolism–related genes, reducing hepatic lipid accumulation, enhancing insulin sensitivity, attenuating inflammation, and improving disease progression. Further validation through Western blotting and RT-PCR confirmed that GLP-2 restored the protein and mRNA expression of AdipoR1/2, AMPK, and PPARα in FFA-treated HepG2 cells (*p*<0.01). Meanwhile, GLP-2 intervention upregulated the secretion level of HMW adiponectin in HepG2 cells (*p*<0.01). These results indicate that GLP-2 activates the ADPN signaling pathway and its downstream mediators, AMPK and PPARα, thereby promoting fatty acid oxidation, inhibiting lipogenesis, and alleviating hepatic steatosis.

ADPN, an adipokine secreted by adipocytes, among the various biologically active forms of adiponectin, HMW adiponectin is the most important and effective subtype that exerts insulin-sensitizing and metabolic protective effects. ADPN regulates multiple metabolic processes through its receptors AdipoR1/R2 and plays a significant role in the occurrence and development of MASLD. ADPN regulates multiple metabolic processes *via* its receptors, AdipoR1 and AdipoR2, and plays a pivotal role in MASLD development and progression [39]. It regulates lipid metabolism, suppresses inflammation, and improves insulin sensitivity through the AMPK and PPARα pathways. Reduced circulating ADPN levels have been consistently identified in epidemiological studies as an independent risk factor for MASLD and impaired liver function [40]. During disease progression, ADPN expression declines by approximately 20–40 % from simple steatosis to steatohepatitis, underscoring its central role in MASLD pathology [41]. AdipoR1 is predominantly expressed in skeletal muscle and liver, whereas AdipoR2 is primarily localized in the liver [42]. AdipoR1 activates AMPK signaling, while AdipoR2 activates PPARα, and together they regulate glucose and lipid metabolism, inflammatory responses, and oxidative stress [43,44].

AMPK serves as a central regulator of cellular energy homeostasis and is activated by elevated intracellular adenosine monophosphate (AMP) and adenosine diphosphate (ADP) levels [45]. Its activation suppresses lipogenesis while enhancing fatty acid oxidation and lipolysis in adipocytes both *in vitro* and *in vivo* [46]. The capacity of AMPK to respond to changes in AMP levels depends on phosphorylation mediated by liver kinase B1 [47]. Evidence indicates that the AMPK/SIRT1 signaling pathway, downstream of AdipoR1, is crucial in reducing hepatocyte lipid deposition by promoting fatty acid oxidation and inhibiting lipogenesis, thereby identifying AdipoR1 as a potential therapeutic target for MASLD [48]. AMPK is essential for regulating energy metabolism and fatty acid oxidation, with a key role in the pathological mechanisms underlying MASLD [49]. Activation of the AMPK pathway mitigates oxidative stress in hepatocytes, decreases excessive reactive oxygen species production, and consequently alleviates lipid accumulation [50–53].

PPARα, a critical regulator of obesity and lipid metabolism, plays a pivotal role in fatty acid metabolism as well as in inflammatory and immune responses. Pharmacological activation of PPARα improves insulin sensitivity, regulates blood lipid and glucose levels, enhances fatty acid catabolism, and reduces triglyceride and cholesterol accumulation, thus having therapeutic relevance in MASLD [54]. Upon activation, AdipoR2 directly binds to and activates PPARα, significantly improving lipid metabolism disorders and liver fibrosis while reducing inflammatory marker levels [55]. ADPN exerts its effects by binding to its receptors, AdipoR1 and AdipoR2, thereby activating the AMPK/PPARα pathway, which promotes fatty acid oxidation and inhibits lipid synthesis. This action preserves hepatic function by increasing insulin sensitivity and preventing triglyceride accumulation, thereby slowing the onset and progression of MASLD [56–58]. The present findings are consistent with these observations, further supporting that GLP-2 improves hepatocyte lipid metabolism disorders by enhancing ADPN activity and its receptor signaling, synergistically modulating the AMPK/PPARα axis. These results provide new insights into the role of GLP-2 in lipid metabolism regulation and the pathogenesis of MASLD.

Our findings on the GLP-2-adiponectin-AMPK/PPARα axis provide a mechanistic explanation for the clinical observations reported in our previous cohort, where GLP-2 was correlated with adverse metabolic parameters [25]. This transition from clinical correlation to mechanistic insight represents a significant advance, yet it is important to acknowledge that the current study focused primarily on lipid metabolism. We did not investigate other potential pathways through which GLP-2 may confer benefits, such as anti-inflammation, improvement of insulin sensitivity, or alleviation of oxidative stress, as suggested by the “multiple-hit” pathogenesis theory of MASLD.

In addition to the focused mechanistic scope, the translational relevance of our findings is constrained by the experimental model. Nevertheless, the use of the HepG2 cell line did not fully replicate the complex pathological microenvironment of MASLD. Future investigations are expected to employ liver-specific AdipoR1 and AdipoR2 knockout mouse models to validate the critical involvement of these receptors in the effects of GLP-2. Additional research will also using the specific antagonist of GLP-2 receptor (GLP-2R) Parazacco spilurus subspand examine the regulatory roles of AMPK and PPARα phosphorylation within the downstream signaling pathway of GLP-2 to improve mechanistic understanding. GLP-2 represents a promising therapeutic target for MASLD through modulation of the AMPK/PPARα signaling axis. However, definitive evidence regarding direct interactions between these pathways remains insufficient and requires validation using approaches such as immunoprecipitation and surface plasmon resonance. Further studies are anticipated to focus on the development of long-acting GLP-2 agonists and to advance clinical translational research to comprehensively assess the therapeutic potential of GLP-2.

## Conclusion

In summary, the findings indicate that GLP-2 synergistically reduced FFA-induced hepatic lipid deposition through modulation of the ADPN/AdipoRAMPK/PPARα signaling axis. The regulation of 701 specific genes, together with dose-dependent effects, provides a molecular basis for the development of GLP-2 analogs targeting MASLD. A novel mechanism was identified in which GLP-2 directly regulated hepatocyte lipid metabolism *via* the AMPK/PPARα pathway, thereby offering a potential therapeutic avenue for MASLD. Future research is required to evaluate the generalizability of this pathway in complex physiological models, such as the HFD mouse model, and to explore the design of long-acting GLP-2 agonists aimed at addressing gut-liver axis signaling dysfunction and systemic metabolic disturbances.

## Figures and Tables

**Table 1:** Primers utilized for qRT-PCR analysis.

*Gene*	Primers	Product size (bp)
*ADIPOR1*	S: ACGGCTCATCTACCTCTCCATCG	113
	A: GGAACACGCCTGCTCTTGTCTG	
*ADIPOR2*	S: ATCGGGGAGTAAGAGCAGGAGTG	138
	A: GCCATCAGCATCAACCAGCCTATC	
*AMPK*	S: CGGCAAAGTGAAGGTTGGCAAAC	96
	A: CCTACCACATCAAGGCTCCGAATC	
*PPARα*	S: TCGGCGAGGATAGTTCTGGAAGC	136
	A: ACCACAGGATAAGTCACCGAGGAG	
*β-actin*	S: GGACTTCGAGCAGGAGATGG	138
	A: AGGAAGGAGGGCTGGAAGAG	

## Abbreviations

AdipoR1: Adiponectin Receptor 1
AdipoR2: Adiponectin Receptor 2
ADPN: Adiponectin
AMPK: AMP-Activated Protein Kinase
DEGs: Differentially expressed genes
FASN: Fatty Acid Synthase
FFAs: Free Fatty Acids
GLP-2: Glucagon-like Peptide-2
GLP-2R: glucagon-like peptide-2 receptor
HFD: high-fat diet
HMW: High-Molecular-Weight
MASH: metabolic dysfunction‐associated steatohepatitis
MASLD: metabolic dysfunction-associated steatotic liver disease
PPARα: Peroxisome Proliferator-Activated Receptor α
